# Impact of Single Nucleotide Polymorphisms (SNPs) on Immunosuppressive Therapy in Lung Transplantation

**DOI:** 10.3390/ijms160920168

**Published:** 2015-08-25

**Authors:** Jesus Ruiz, María José Herrero, Virginia Bosó, Juan Eduardo Megías, David Hervás, Jose Luis Poveda, Juan Escrivá, Amparo Pastor, Amparo Solé, Salvador Francisco Aliño

**Affiliations:** 1 Unidad de Farmacogenética, Instituto de Investigación Sanitaria La Fe, Hospital Universitario y Politécnico La Fe, Av. Fernando Abril Martorell 106, 46010 Valencia, Spain; E-Mails: jrzrms@gmail.com (J.R.); boso_vir@gva.es (V.B.); megias_jua@gva.es (J.E.M.); salvador.f.alino@uv.es (S.F.A.); 2 Servicio de Farmacia, Hospital Universitario y Politécnico La Fe, Av. Fernando Abril Martorell 106, 46010 Valencia, Spain; E-Mail: poveda_josand@gva.es; 3 Departamento Farmacología, Facultad de Medicina, Universidad de Valencia, Av. Blasco Ibáñez 15, 46010 Valencia, Spain; 4 Unidad de Bioestadística, Instituto Investigación Sanitaria La Fe. Av. Fernando Abril Martorell 106, 46010 Valencia, Spain; E-Mail: bioestadistica@iislafe.es; 5 Unidad de Trasplante Pulmonar, Hospital Universitario y Politécnico La Fe, Av. Fernando Abril Martorell 106, 46010 Valencia, Spain; E-Mails: escriva_juan@gva.es (J.E.); pastor_ampcol@gva.es (A.P.); sole_amp@gva.es (A.S.); 6 Unidad de Farmacología Clínica, Área Clínica del Medicamento, Hospital Universitario y Politécnico La Fe, Av. Fernando Abril Martorell 106, 46010 Valencia, Spain

**Keywords:** pharmacogenetics, lung transplantation, tacrolimus, mycophenolic acid, *P*-glycoprotein

## Abstract

Lung transplant patients present important variability in immunosuppressant blood concentrations during the first months after transplantation. Pharmacogenetics could explain part of this interindividual variability. We evaluated SNPs in genes that have previously shown correlations in other kinds of solid organ transplantation, namely *ABCB1* and *CYP3A5* genes with tacrolimus (Tac) and *ABCC2*, *UGT1A9* and *SLCO1B1* genes with mycophenolic acid (MPA), during the first six months after lung transplantation (51 patients). The genotype was correlated to the trough blood drug concentrations corrected for dose and body weight (C_0_/D_c_). The *ABCB1* variant in rs1045642 was associated with significantly higher Tac concentration, at six months post-transplantation (CT *vs.* CC). In the MPA analysis, CT patients in *ABCC2* rs3740066 presented significantly lower blood concentrations than CC or TT, three months after transplantation. Other tendencies, confirming previously expected results, were found associated with the rest of studied SNPs. An interesting trend was recorded for the incidence of acute rejection according to *NOD2/CARD15* rs2066844 (CT: 27.9%; CC: 12.5%). Relevant SNPs related to Tac and MPA in other solid organ transplants also seem to be related to the efficacy and safety of treatment in the complex setting of lung transplantation.

## 1. Introduction

Lung transplantation is the option for a variety of end-stage pulmonary diseases. However, it remains as the solid organ transplant with the poorest outcomes, with less than 80% and 70% survival after one and three years, respectively [1]. Several reasons for these poor results have been identified, some of them being shared with other solid organ transplants, such as acute rejection and drug treatment toxicity [2,3].

Currently, solid organ transplant therapy includes treatment with broad-spectrum immunosuppressive regimens, usually calcineurin antagonists (cyclosporin (Cs) and tacrolimus (Tac)) together with mycophenolic acid (MPA). These drugs have a narrow therapeutic index and important inter-individual variability in their pharmacokinetic profile. Therefore, patients subjected to such immunosuppressive therapy are at risk of under-immunosuppression, potentially leading to graft loss or, contrarily, excessive drug concentrations potentially leading to multiple drug toxicities such as hematologic toxicity or renal failure. Identifying the contributing factors that account for variation in blood drug concentrations is of major importance in these patients and can be used to optimize therapeutic strategies and increase the benefit—Risk ratio of immunosuppressive therapy.

Over the last decade there has been a growing interest in genetic variability based on single nucleotide polymorphisms (SNPs) and their effects on immunosuppressive therapy in transplant patients. Among the different SNPs studied, those associated with the genes that encode for *P*-glycoprotein (*P*-gp) and for CYP3A enzymes have generated special interest. Calcineurin inhibitors are substrates for the membrane transporter *P*-gp (*ABCB1* gene), and genotypes involving greater *ABCB1* function have been associated with poorer drug absorption and lower concentrations/doses. *CYP3A* corresponds to a superfamily of enzymes involved in the metabolism of many drugs, including Cs and Tac. Therefore, genotypes that inhibit or increase the activity of these enzymes would alter the blood concentration of these drugs [4]. Genetic polymorphisms in enzymes involved in MPA transport (*ABCC2*, *SLCO1B1*) and metabolism (*UGT1A9*) have also been studied, though contradictory results regarding their effects have been reported [5,6,7,8,9].

Lung transplantation patients are a particularly difficult group to study. Immunosuppressive treatment variations and the way in which such treatment is administered (via the intravenous and oral routes) during the first weeks post-transplantation, make changes in drug blood concentration difficult to evaluate. As a consequence of such complexity, this group of patients has been less studied than other groups such as heart, liver or kidney transplant patients [10].

Given the limited information available in this group of patients, we decided to investigate the relationship between blood Tac and MPA concentrations and the presence of certain adverse events with some SNPs that have already been related to important variations in immunosuppressive therapy in other solid organ transplantations [11,12,13,14,15]. Our aim was to confirm that those SNPs described to be potentially meaningful in the clinical setting (according to the literature and results obtained by our group) in other solid organ transplanted patients, show the same behavior in the complex context of lung transplantation.

## 2. Results and Discussion

The advances made in recent years have caused lung transplantation to become essential treatment option for some irreversible lung diseases. Post-transplantation drug monitoring aims to maintain optimal immunosuppression for each specific patient, maximizing pharmacological efficacy while minimizing toxicity. However, the pharmacokinetics of Tac and MPA are characterized by important inter-patient variability and poor bioavailability. This situation is particularly severe in lung transplantation patients, where high variability in drug concentrations has been reported [16]. Identifying genetic variants responsible for the wide variability of concentrations in these patients is essential in order to achieve treatment success. The aim of this work was to try to confirm in our series of lung transplanted patients some relationships between certain SNPs and immunosuppressant drug pharmacokinetics and pharmacodynamics previously reported by our own group. Our main limitation has been the small number of patients that we have been able to include: the results therefore must be interpreted with caution. The hypothesis-driven approach is statistically correct when working with small sample sizes as in the case of our cohort.

**Table 1:** Characteristics of the study subjects (*n* = 51).

Gender (Male/Female)	34/17
Age at transplant (Mean (SD))	45.4 (16.3)
Type of lung transplantation (Single/Double/Heart-Lung)	(17/32/2)
**Primary indication of lung transplantation, no. (%)**	
COPD ^1^, including emphysema	15 (29.4)
Cystic fibrosis	13 (25.5)
Idiopathic pulmonary fibrosis	16 (31.3)
Others	7 (13.8)

^1^ COPD: chronic obstructive pulmonary disease.

The baseline characteristics of our study population are shown in Table 1. The genotype frequencies obtained for each tested SNP were consistent with those described in public databases for the Caucasian population in most cases (Table 2). Some of them have been previously described as significantly different in our Spanish population subset [17].

**Table 2:** Frequency of genotypes of the SNPs included in the study (*n* = 51 patients).

Gene	SNP	Variant	Frequency (%)	Expected Frequency (%) *
ABCB1	rs1045642 (3435 C > T)	CC	39.2	27.1
		CT	35.3	51.8
		TT	25.5	21.1
	rs1128503 (1236 T > C)	CC	47.0	32.5
		CT	37.3	49.8
		TT	15.7	17.7
	rs2032582 (2677 G > T/A)	GG	45.1	35.1
		GT	50.1	50.8
		TT	4.8	12.1
	rs9282564 (61 A > G)	AA	90.2	81.7
		AG	9.8	16.7
		GG	0.0	1.7
ABCC2	rs3740066 (3972 C > T)	CC	41.2	58.3
		TC	41.2	35.7
		TT	17.6	6.0
	rs2273697 (1249 G > A)	GG	64.7	57.6
		GA	33.3	35.3
		AA	2.0	7.1
	rs717620 (-24 C > T)	CC	54.9	56.7
		CT	41.2	41.6
		TT	3.9	1.7
CYP3A5	rs776746 (CYP3A5*3)	GA	96.1	83.3
		GA	3.9	15.7
		AA	0.0	1.1
NOD2/CARD15	rs2066844 (R702W)	CC	84.3	87.7
		CT	15.7	11.8
		TT	0.0	0.5
SLCO1B1	rs2306283 (SLCO1B1*1B)	AA	43.7	40.1
		AG	41.7	43.7
		GG	15.6	16.2
	rs4149056 (SLCO1B1*5)	TT	74.5	70.0
		TC	19.6	28.3
		CC	5.9	1.7
UGT1A9	rs6714486 (-275T > A)	TT	88.2	85.0
		TA	11.8	15.0
		AA	0.0	0.0
	rs17868320 (-2152 C > CT)	CC	100.0	98.2
		CT	0.0	1.2
		TT	0.0	0.0

* According to the Caucasian population (“dbSNP” database, http://www.ncbi.nlm.nih.gov/snp, accessed on March 2015).

### 2.1. Tacrolimus Analysis

**Figure 1:**
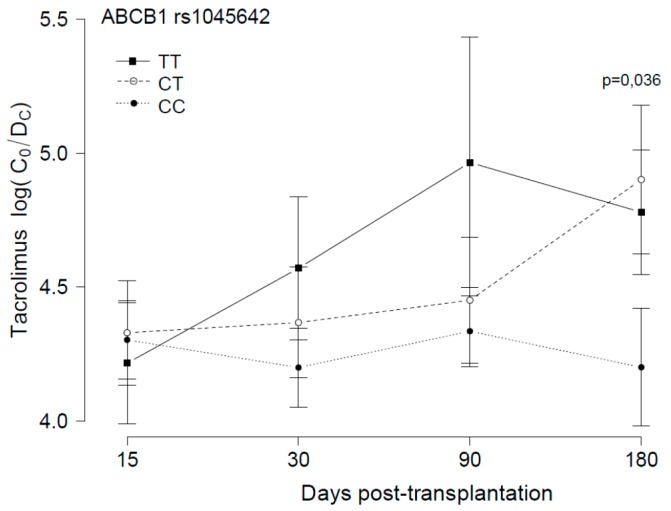
Tacrolimus blood concentration in relation to the *ABCB1* rs1045642 genotype. Tacrolimus corrected (dose/weight) trough concentrations (C_0_/D_c_, [(ng/mL)/(mg/kg/24 h)]) are plotted (mean and standard deviation of all the measurements in each period) in relation to the *ABCB1* rs1045642 genotype of 40 lung transplant patients, during the first six months post-transplantation.

An association between *ABCB1* SNPs and blood Tac concentration was found in the rs1045642 analysis. Higher blood concentrations, expressed as trough blood drug concentrations corrected for dose and body weight (C_0_/D_c_), were associated to the T allele, comparing CT with TT and CC patients. Significant differences were found between Tac concentrations in CT *versus* CC patients, six months after transplantation (*p* = 0.036) (Figure 1).

**Figure 2:**
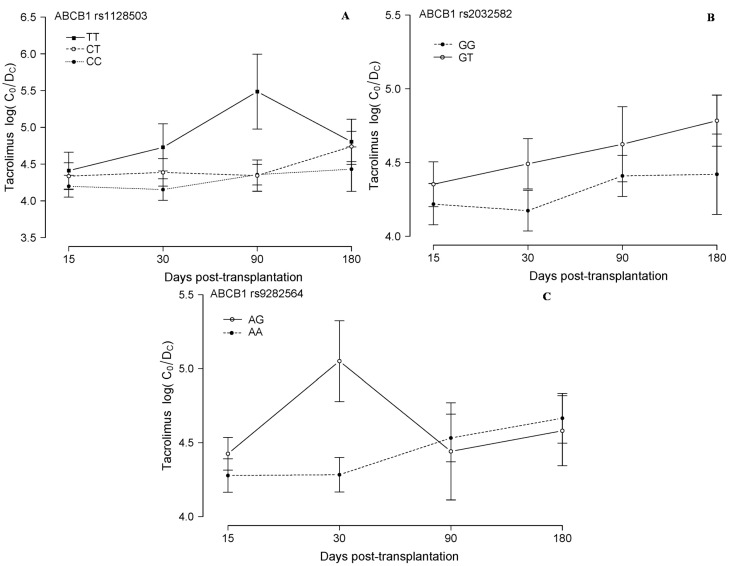
Tacrolimus blood concentration in relation to other *ABCB1* SNPs. Tacrolimus corrected (dose/weight) trough concentrations (C_0_/D_c_, [(ng/mL)/(mg/kg/24 h)]) are plotted (mean and standard deviation of all the measurements in each period) in relation to the *ABCB1* genotype of 40 lung transplanted patients, during the first six months post-transplantation, according to rs1128503 in (**A**), to rs2032582 in (**B**) and to rs9282564 in (**C**).

Regarding the rs1128503 analysis, the presence of the T variant was also associated with higher adjusted blood Tac levels from the first to the sixth month post-transplantation, though significant differences were not observed (Figure 2A). The third most relevant SNP in the *ABCB1* gene, rs2032582, also showed the expected behaviour according to the literature and our previous data in other series of solid organ transplant patients: the T allele showed a tendency to be associated to increasing blood Tac levels (Figure 2B) [18,19,20], though here again the data did not reach statistical significance, and in this case none of our patients had the TT genotype.

There is a SNP in the *ABCB1* gene, rs9282564, comprising the change Asn21Asp, due to the variation A > G, the biological effect of which has not been clearly established. One study reported that *in vitro*, no differences were found between the variants regarding the efflux ability of *P*-gp, except for one of several tested drugs [21]. However, in our own experience, we have recently described that the G variant correlates to significantly increased blood Cs concentration two months after transplantation in heart recipients, correlating also to lesser renal function [22]. The data from our lung transplant patients also support this observation, as shown in Figure 2C, where no statistically significant results are obtained, but where an increase in blood Tac concentration is observed at least during the first post-transplantation month, in patients carrying the G variant (only heterozygous individuals in this series of patients).

The SNP rs776746 in the *CYP3A5* gene has been postulated as the main pharmacogenetic polymorphism for predicting differences in patient blood Tac levels [13,18,23,24]. This has even been supported by meta-analyses [25,26], and so far it has been the only SNP considered in a published prospective randomized study that assigned different Tac starting doses according to the recipient genotype [27]. Recently, even one of the two main international consortia working in pharmacogenetics translation to clinical practice, the Clinical Pharmacogenetics Implementation Consortium (CPIC) has published dosing guidelines for tacrolimus according to *CYP3A5* SNPs [28]. Unfortunately, in this study, only one patient presented the variant allele (GA), and therefore the relationship between rs776746 and blood Tac concentrations could not be evaluated.

*ABCB1* (multi-drug resistance gene, *MDR1*) is the gene that encodes for *P*-gp, which is one of most important transporters expressed at the apical membrane of cells in multiple tissues. This protein has been associated with alterations in drugs disposition by reducing or increasing absorption. Several studies on *ABCB1* polymorphisms have reported the association between *P*-gp expression and function and tacrolimus pharmacokinetics [29,30,31]. However, contradictory results have been reported regarding the effect of *ABCB1* polymorphisms on tacrolimus disposition in lung transplantation. *Wang et al*., in 91 patients, found an association between the *ABCB1* haplotype and blood Tac concentration [32], in agreement with the results obtained in our patients. However, other studies have found no such association [33,34]. In these studies no consideration was made of the concomitant effect of the *CYP3A5* genotype, which has been shown to have important effects [35]. In our study, we only had one patient with the *CYP3A5* SNP variant, so we could confirm the previously reported effects of the three *ABCB1* SNPs, but not of that corresponding to *CYP3A5*.

**Figure 3:**
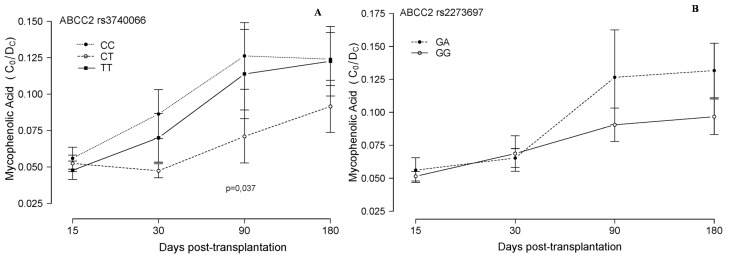
Mycophenolic acid blood concentration in relation to *ABCC2* SNPs. Mycophenolic acid corrected (dose/weight) trough concentrations (C_0_/D_c_, [(ng/mL)/(mg/kg/24 h)]) are plotted (mean and standard deviation of all the measurements in each period) in relation to the *ABCC2* rs3740066 (**A**) and the *ABCC2* rs2273697 (**B**) genotypes of 51 lung transplanted patients, during the first six months post-transplantation.

### 2.2. Mycophenolic Acid Analysis

To our knowledge, no previous studies correlating SNP variations and MPA concentrations specifically in lung transplantation have been published. However, relationships have been previously studied in other kinds of patients. In renal and paediatric heart transplant patients, polymorphisms of *UGT1A9* and *ABCC2* have been associated with variations in plasma levels of MPA [9,36,37] and adverse events [38,39,40]. Trough concentration monitoring has been described to be a poor indicator of MPA exposure and this is a limitation in our study. Nevertheless, it is the parameter that is routinely measured in many hospitals, so we have tried to determine whether the associations already described also exist in our series of patients [41]. In the present study we have evaluated the impact of *ABCC2* rs717620, rs3740066 and rs2273697 upon blood MPA levels. Heterozygous patients (CT) at *ABCC2* rs3740066 had significantly lower blood MPA concentrations than homozygotes (CC and TT) (*p* = 0.037, Figure 3A) three months after transplantation. The published data regarding the effects of *ABCC2* SNPs are inconclusive, though many efforts have been made to try to elucidate the biological meaning of variants in this gene. We found higher blood drug levels with TT but also with CC genotypes, compared to CT, which were always lower. This phenomenon, known as heterozygote advantage, has not been previously described for this SNP, whose biological effect is in fact controversial, but other examples have been published in very well characterized SNPs, such as for instance the *MTHFR* gene [42,43]. We need to continue including more patients in order to confirm these results.

**Figure 4:**
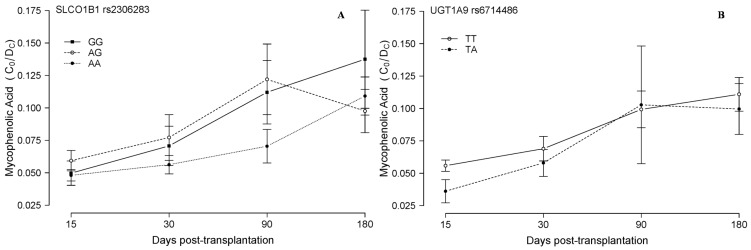
Mycophenolic acid blood concentration in relation to other associated SNPs. Mycophenolic acid corrected (dose/weight) trough concentrations (C_0_/D_c_, ((ng/mL)/(mg/kg/24 h))) are plotted (mean and standard deviation of all the measurements in each period) in relation to the *SLCO1B1* rs2306283 (**A**) and the *UGT1A9* rs6714486 (**B**) genotypes of 51 lung transplanted patients, during the first six months post-transplantation.

In relation to rs2273697, the literature again reports controversial effects. According to our data (Figure 3B), and although statistical significance was not reached, GA patients showed higher blood MPA concentration than GG patients in the later periods analyzed, *i.e.*, at 90 and 180 days post-transplantation. This would be in agreement with published works [44].

Regarding the influx MPA transporter *SLCO1B1*, two common non-synonymous variants have been well characterized: rs2306283 (encoding for N130D) and rs4149056 (encoding for V174A). These two variants are in partial linkage disequilibrium, and have been widely studied in other settings, especially in statins therapy. In our patients, *SLCO1B1* rs2306283 patients carrying the AA genotype seemed to be associated with lower blood MPA concentration, compared to GG and AG, practically during the six-month follow-up period, though no significant differences were reached (Figure 4A). Moreover, *SLCO1B1* has been associated with the presence of adverse events [45], which have also been associated with high MPA concentrations [46].

In the study of the SNPs in the metabolizing enzyme *UGT1A9*, one of them, rs17868320, was monomorphic in our series of patients. The other *UGT1A9* SNP, rs6714486, showed the expected behavior according to previous data: TT patients showed higher MPA levels than TA patients, though again statistical significance was not reached (Figure 4B). Finally, both rs4149056 in *SLCO1B1* and rs717620 in *ABCC2* showed no remarkable differences in blood drug levels, unlike the rest of the SNPs evaluated (data not shown). These also could be relevant SNPs and have been extensively studied in the literature. More in depth studies therefore will be needed, with more patients and including confounding covariates (cystic fibrosis, other SNPs, *etc.*), in order to understand the real meaning of these SNPs in the lung transplantation setting.

### 2.3. Adverse Events

Unfortunately, we could not confirm any of the hypotheses regarding an association between our evaluated SNPs and the reported adverse events retrieved. In any case, although no significant differences were obtained, we found that the *NOD2/CARD15* polymorphism may play a role in the presence of acute rejection in these patients. We recorded a 27.9% acute rejection rate associated with the CT genotype compared with 12.5% in relation to the TT genotype at rs2066844. These results are in agreement with those obtained from other studies, including our own, where polymorphisms in this gene influence the organ survival outcomes of transplant patients [20,47,48]. Due to the potential relevance of this finding, we think that this gene should be included in future studies including more patients.

The main limitations of our study include the small number of patients involved (*n* = 51), the high dispersion of blood concentration values, and the difficulties in analyzing blood drug concentrations when Cs and Tac have both been administered in the same patient, even through different administration routes (oral and intravenous). Although seeking to identify an association between adverse effects and polymorphisms in such a small cohort is unrealistic, we decided to analyze the SNP rs2066844 in NOD2/CARD15, according to our previous results [22]. All these limitations may have prevented the finding of significant differences, though clear tendencies were seen in most of the analyzed situations.

The influence of different genetic polymorphisms on plasma immunosuppressant concentrations and toxicity has been the subject of numerous publications lasting recent years. However, the implementation of pharmacogenetics in transplantation medicine has not met the expectations expressed years ago [49]. The results obtained in this study support the idea of using pharmacogenetic analysis prior to lung transplantation in order to help obtain success in transplantation and preserve patient safety. Other SNPs of genes not included in our study, have shown relevant influence on the immunosuppressants, as CYP3A4 regarding Tac and Cs [50,51]; and IMPDH1 and 2 regarding MPA [52,53]. The implementation of these variants in our study could be of great value. It will also be of fundamental importance to analyze the SNPs related to each patient treatment as a whole, *i.e.*, including all the concomitant drugs, not only the immunosuppressants administered together, but also other drugs, such as for instance, omeprazole [54]. This is the only way to understand patient response and apply the necessary therapeutic decisions.

## 3. Experimental Section

We designed a single-center retrospective study to genotype SNPs previously associated with blood Tac and MPA concentrations, in order to confirm their association in our series of lung transplant patients. Fifty-one Caucasian patients who received tacrolimus (*n* = 40) and/or MPA (*n* = 51) after lung transplantation from January 2008 to January 2011, and provided written informed consent were included.

Patients were followed-up on for the first six months after transplantation for the pharmacokinetic and clinical data. All the recruited patients were Caucasian. Data were obtained retrospectively from the medical records.

Genetic data and any other relevant information were retrieved from the study population without interfering with patient treatment and were handled according to standard regulations referred to data registration, use, and the preservation of patient anonymity and privacy. The study was approved by the local Clinical Research Ethics Committee (registry number 2008/0263), and was conducted in accordance with the Declaration of Helsinki (Istanbul version and its amendments).

### 3.1. Blood Immunosuppressants Concentration

Immunosuppressive drug concentrations were measured in whole blood samples collected immediately before morning dose administration (C_0_, trough level, in ng/mL) using clinical chemistry systems (Dimension^®^; Siemens Healthcare, Deerfield, IL, USA, for tacrolimus advanced chemiluminescence immunoassay and Viva-E^®^; Siemens Healthcare, for MPA enzyme multiplied homogeneous immunoassay). The mean and standard deviation of dose-normalized blood concentrations during each period (first 15, 30, 90 and 180 days after transplantation) were expressed as the trough concentration/dose corrected for weight ratio (C_0_/Dc) ((ng/mL)/(mg/kg/24 h)).

### 3.2. Genotyping

Genomic DNA was collected from EDTA-anticoagulated whole blood samples from transplant recipients. The DNA was extracted from 200 mL of blood with the use of a commercially available kit based on centrifugation in microcolumns (Ultra Clean Blood Spin DNA Isolation kit; MoBio Laboratories Inc.: Carlsbad, CA, USA). After quantification using a spectrophotometer (NanoDrop Technologies Inc.: Wilmington, DE, USA) to determine concentration and purity, the DNA was stored at −20 °C until use. A genetic analysis platform (Mass ARRAY; Sequenom, Inc.: San Diego, CA, USA) was used to genotype each sample.

The relation to blood immunosuppressant concentrations was investigated in 12 SNPs from 5 different genes: *ABCB1* (rs1045642, rs1128503, rs2032582, rs9282564) and *CYP3A5* (rs776746) for Tac, and *ABCC2* (rs2273697, rs3740066, rs717620), *SLCO1B1* (rs2306283, rs4149056) and *UGT1A9* (rs6714486, rs17868320) for MPA.

### 3.3. Clinical Outcomes

Occurrence of acute rejection, serious infections and loss of renal function was documented from medical records and associations with *NOD2/CARD15* rs2066844 SNP and *UGT1A9* rs6714486 for the first; *ABCB1* rs1128503 for the second; and *ABCB1* rs9282564 for the third, respectively, were assessed, according to previously published findings [22].

### 3.4. Statistical Analysis

Data were summarized as the mean and standard deviation in the case of continuous variables and as relative and absolute frequencies in the case of categorical variables. Associations between blood drug concentration and the different SNPs were assessed using mixed linear regression models with time, genotype and their interaction as predictors and drug levels (log-transformed in the case of Tac) as response variable. The individual was added as a random factor to account for the repeated measures design. Association with graft rejection, infection and renal dysfunction was assessed using logistic regression models. A *p*-value < 0.05 was considered statistically significant. All statistical analyses were performed with R (version 3.1.2) and R-package lme4 (version 1.1–7).

## 4. Conclusions

In our hands, blood Tac concentrations in lung transplant patients are affected by *ABCB1* polymorphisms, with significant differences in relation to rs1045642, being evident from treatment initiation until three months post-transplantation. Analogously, blood MPA concentrations are related to *ABCC2*, *UGT1A9* and *SLCO1B1* SNPs, reaching statistical significance in relation to *ABCC2* rs3740066. In this study we have tested the most relevant SNPs related to the transport and metabolism of Tac and MPA in lung transplant patients, confirming the results expected (though mainly in the form of non-significant tendencies) according to the literature and our own data in other solid organ transplant patients. This confirms the potential utility of these SNPs in the complex setting of lung transplantation. Further studies involving larger numbers of patients are needed to confirm these results.
